# Psychosocial determinants of sleep difficulties in adolescence: the role of perceived support from family, peers, and school in an Italian HBSC sample

**DOI:** 10.1007/s00431-023-04934-0

**Published:** 2023-03-23

**Authors:** Ilaria Maria Antonietta Benzi, Silvano Gallus, Eugenio Santoro, Lavinia Barone, the HBSC Lombardy Committee, Franco Cavallo, Liliana Coppola, Corrado Celata, Antonella Delle Fave, Elisabetta Nigris, Luca Vecchio, Marco Terraneo, Mara Tognetti, Lavinia Barone, Silvia Salvatore, Stefano Capolongo, Elena Marta, Edoardo Lozza, Aleksandra Torbica, Vincenzo Russo, Silvano Gallus, Eugenio Santoro, Lucia Crottogini, Claudia Lobascio, Mariacira Veneruso, Giusi Gelmi, Chiara Scuffi, Veronica Velasco, Giuliana Rocca, Paola Ghidini, Ornella Perego, Raffaele Pacchetti, Maria Stefania Bellesi, Silvia Maggi, Elena Nichetti, Antonella Giannellini, Federica Di Cosimo, Mariacira Veneruso, Davide Montani, Marina Ghislanzoni, Carla Torri, Elena Scarpanti, Laura Stampini, Cosimo Scaglione, Angela Sacchi, Marcella Linda Casalini

**Affiliations:** 1 Department of Brain and Behavioral Sciences, University of Pavia, Piazza Botta Adorno Antoniotto, 11, 27100 Pavia, Italy; 2 Department of Medical Epidemiology, Istituto di Ricerche Farmacologiche Mario Negri IRCCS, Milan, Italy; 3 Laboratory of Medical Informatics, Department of Public Health, Istituto di Ricerche Farmacologiche Mario Negri IRCCS, Milan, Italy

**Keywords:** Adolescence, Sleep difficulties, Health Behavior in School-Aged Children (HBSC), Family support, Peer support

## Abstract

The present study explores the concurrent contribution to sleep problems of individual-related, family-related, and school-related factors in adolescence. Gathering from the Italian 2018 Health Behavior in School-Aged Children (HBSC) data collection, we used hierarchical logistic regression on a sample of 3397 adolescents (51% females, Mage = 13.99, SD = 1.62) to explore the contribution to sleep problems of the individual (Model 1: alcohol use, smoking, screen time, physical activity), familial (Model 2: parental communication, parental support), and school-related (Model 3: peer support, schoolmates/students support, teacher support and school pressure) variables. 28.3 percent of adolescents reported having sleep difficulties. Overall, Model 3 significantly improved over Model 2 and Model 1. Data showed that increasing smoking (OR = 1.11; 95% CI: 1.03–1.20) and screen time (OR = 1.05; 95% CI: 1.02–1.08) were associated with sleep difficulties but not alcohol use and physical activity. Also, impaired communication with both parents and increasing parental support (OR = 0.84; 95% CI: 0.78–0.90) were associated with decreased odds of sleep problems. Finally, both increases in school pressure (OR = 1.40; 95% CI: 1.26–1.56) and lack of student support (OR = 1.25; 95% CI: 1.10–1.42) were associated with a higher likelihood of sleep problems, while peer support and teacher support were not.

*Conclusion*: Our findings highlight the importance of an integrated approach to the study of sleep difficulties in adolescence that includes specific psychosocial contributors such as the quality of parental communication and perceived parental support and considers the quality of the day-to-day relationship with schoolmates and the school level of demands.

**Table:** 

**What is Known:** *• Adolescents' are at-risk of more significant sleep difficulties, and recent literature highlights the importance of an integrated approach to understanding this phenomenon, including biological, psychosocial, and contextual factors.* • *The literature lacks findings that consider the concurrent contribution of individual and psychosocial factors to sleep difficulties in adolescence*.	
**What is New:** • *The quality of parental communication and perceived parental support, as expressions of adult figures' emotional and behavioural availability in the adolescent's life, are significant determinants of sleep difficulties*. •* The quality of day-to-day relationships with schoolmates and the school level of demands contribute to adolescent sleep problems*.

**What is Known:**

*• Adolescents' are at-risk of more significant sleep difficulties, and recent literature highlights the importance of an integrated approach to understanding this phenomenon, including biological, psychosocial, and contextual factors.*

• *The literature lacks findings that consider the concurrent contribution of individual and psychosocial factors to sleep difficulties in adolescence*.

**What is New:**

• *The quality of parental communication and perceived parental support, as expressions of adult figures' emotional and behavioural availability in the adolescent's life, are significant determinants of sleep difficulties*.

•* The quality of day-to-day relationships with schoolmates and the school level of demands contribute to adolescent sleep problems*.

## Introduction

### Sleep difficulties in adolescence

Sleep is a critical element for human health and well-being. Moreover, the literature has shown a negative impact of sleep-related problems on physical and psychological health [1]. These considerations are even more important in adolescence. Indeed, during this developmental period, good sleep quality can contribute to healthy brain development (which involves the brain's frontal regions until early adulthood) and overall well-being [2, 3]. However, research shows that youths are at risk for lower sleep quality during adolescence. For example, a recent epidemiological study highlighted that Italian adolescents are particularly at risk for insufficient sleep, restless sleep and difficulty falling asleep [4]. Indeed, changes in circadian rhythms typical of this developmental period and the impact of variables that contribute to decreased sleep-time availability (i.e., early school hours) contribute to insufficient sleeping hours. In addition, studies have shown a frequent bi-directional relationship between inadequate, or lack of, sleep and physical and psychological problems contributing to vicious cycles that negatively impact the quality of adolescents' life [5]. Indeed, several contributions found significant associations between adolescents’ sleep problems and cognitive, behavioral, emotional, and functional consequences (i.e., school performance) [6, 7]. Overall, contemporary research has emphasized the importance of adopting an integrated model for understanding sleep difficulties in adolescence: biological, psychosocial, and contextual factors influence each other nonlinearly and can help elucidate protective and risk factors [8, 9].

Many studies focused on the relationship between individual factors related to unhealthy behaviors, such as alcohol use and smoking, to understand the etiology of sleep problems. For example, literature on alcohol highlights that alcohol contribute to worsening sleep problems in a developmental period were youths are already prone to a lack of sleep as their increasing desire to go to sleep later is combined with early mornings’ school-related commitments; also, available findings show that sleep difficulties contribute to increased alcohol use in youths [10]. Similar patterns were found for smoking [8]. Another well-known maladaptive individual factor is screen time. Numerous contributions in the literature report excessive time in front of a computer or smartphone screen as an essential predictor of sleep difficulties [11]. For example, a recent study of adolescents in 2002, 2006, 2010, and 2014 waves of the Health Behavior in School-Aged Children (HBSC) study emphasized the importance of considering screen time exceeding two hours daily as critical in fostering sleep problems [12]. Finally, another potentially unhealthy subjective behavior is physical inactivity. Again, available literature provides mixed findings as some contributions suggest that physical activity might promote good sleep and, at the same time, others found no associations between physical activity and sleep difficulties [12–14].

### The impact of social connections and perceived support

The recent pandemic has further highlighted the importance of sleep in adolescence, pointing to the extent of considering, together with individual behaviors, also psychosocial factors that may contribute to sleep difficulties: lack of routines, the presence of external stressors, and absence of adequate support from crucial stakeholders in the adolescents' relational universe, such as family and school [15].

Adolescents' relationship quality with the family has been extensively studied. For example, research has emphasized the importance of good communication between parents and adolescents and overall perceived family support. Indeed, both aspects are protective factors for the enactment of risk behaviors (i.e., drugs, alcohol), antisocial behaviors (i.e., bullying, cyberbullying), and general psychological (i.e., depression, anxiety, suicidality) and behavioral adjustment [16–19]. Available literature highlights that a low-stressor family environment, parental warmth, rule-setting, and cohesive relationships all contribute to fewer sleep problems. However, recent contributions highlight the importance of providing more findings on the relationship between family-related variables and adolescents' quality of sleep as available evidence is still scarce [9].

Another essential dimension during this developmental period is school. Indeed, in this context, adolescents can test themselves as future young adults by developing their identity, cultivating meaningful relationships, and evolving personal goals and projects. In this scenario, research has shown how perceived support from teachers and schoolmates influences youths' sense of belonging, engagement, self-esteem, and motivation [20, 21]. For example, a study found that when adolescents perceive teachers to be supportive (i.e., acknowledging emotions and fostering personal development), there is an improvement in their overall well-being [22]. Another contribution showed that in bully/victim groups, greater perceived peer social support contributed to lower anxiety and depressive symptoms [23]. Available contributions point to the importance of considering the effect of loneliness, peer victimization, attachment style, and the amount of time spent with peers: remarkably, evidence on the association between the quality of peer relationships and sleep disturbances is still limited [24–27].

### Aims of the study

Besides one previous study [28], the literature lacks findings that consider the concurrent contribution of individual and psychosocial factors to sleep difficulties in adolescence. Thus, the present study aims at addressing this gap by exploring a representative sample of Italian adolescents from the 2018 Health Behavior in School-Aged Children (HBSC) data collection from Lombardy (northern Italy). This region accounts for one-sixth of the national population, with a size comparable to Austria and Sweden [29].

More specifically, we aim to explore both the individual and concurrent contribution to sleep problems of individual-related (i.e., unhealthy behaviors), family-related (i.e., parental communication, perceived support), and school-related (i.e., perceived support from peers, schoolmates/students, and teachers) factors.

First, based on available research on individual-related factors [7], we expect increasing unhealthy behaviors such as alcohol use, smoking, and screen time to enhance the odds of sleep difficulties. Moreover, in line with previous studies [12], we hypothesize that physical activity will not be associated with adolescents' sleep problems. Second, as suggested by previous research [30], we expect any disruption in parental communication and a decrease in perceived parental support to be associated with sleep difficulties. Third, based on the above evidence [8], we posit that perceived support from peers, schoolmates/students, and teachers will promote having fewer sleep difficulties. At the same time, school pressure will increase the odds of having sleep problems.

Overall, we hypothesize that considering the simultaneous contribution of individual, familial, and school-related factors will provide a more comprehensive clarification of the determinants of sleep difficulties in adolescence.

## Methods

### Participants and procedure

We used Lombardy regional data from the 2018 wave of the HBSC survey.

The HBSC survey is an ongoing World Health Organization collaborative study currently conducted in 51 countries and regions that explores adolescents' health and well-being. The HBSC population comprises adolescents aged 11, 13, and 15 years old enrolled in Italian middle and high schools. To ensure sufficient statistical power and obtain robust frequency estimates at a regional level, schools from the Lombardy region were oversampled [11]. A stratified cluster sampling method was employed to obtain representative samples for each age group, with school classes serving as the primary sampling unit. The complete and alphabetically ordered list of public and private schools in Lombardy was utilized to implement the sampling strategy. The sampling methodology and data collection procedures were designed to ensure that the sample was representative of the adolescent population in Lombardy and to enhance the generalizability of the study findings. Comprehensive details on the sampling methodology and data collection procedures used in the HBSC survey are reported elsewhere.

A significant proportion of adolescents aged 11, 13, and 15: 227,441 young people participated in the 2017/2018 survey [31, 32]. 3397 participants (51% females) from the Italian HBSC data collection from Lombardy who self-reported sleep difficulties were included in the study (99.97% of the original sample). The sample (Mage = 13.99, SD = 1.62) included a 11-year-old class group (N = 1087, 49.31% females), a 13-year-old class group (N = 1173, 48.59% females), and a 15-year-old class group (N = 1137, 55.23% females) of adolescents. The HBSC survey protocol was approved by the Institutional Ethical Board of the National Institute of Health (General protocol: PRE-876/17).

### Measures

#### Outcome variable

Adolescents' sleep difficulties were assessed using a single item ("Over the past six months, how often did you experience difficulties in falling asleep?"), from the HBSC symptoms checklist (HBSC-SCL). The HBSC-SCL is a self-report 8-items scale that assesses health complaints (i.e., headache, abdominal pain, backache, feeling low, irritability or bad mood, feeling nervous, sleeping difficulties and dizziness) on a 5-points Likert scale: 1 = every day, 2 = more than once a week, 3 = almost once a week, 4 = almost once a month, 5 = rarely or never. For this study, a binary outcome was created as a sum of "frequent difficulties" (1 = every day or more than once a week) and "little or no difficulties" (0 = circa once a week to rarely or never) [33].

### Control variables

Control variables included *sex*, *age group*, and demographic variables such as *family structure* ("mother and father", "only mother", "only father", "blended family", "other"), *siblings* ("only child", "one sibling", "at least two siblings"). *Socio-economic status* assessed by the Family Affluence Scale ("low", "medium", "high") (FAS) [34]: the scale consists of six items, including the number of cars, having one's own bedroom, the number of computers, the number of bathrooms, having a dishwasher, and the number of family vacations abroad. The responses are scored from 0–2, and the total score ranges from 0–10. Moreover, we controlled for *self-perceived health* ("excellent", "good", "fair", "poor"), to control for the psychological effect of any illnesses.

### Individual-related variables

Individual-related variables included *smoking* in the last 30 days (measured on a Likert scale from 1 = "never" to 7 = "every day"), *alcohol use* in the previous 30 days (measured on a Likert scale from 1 = "never" to 7 = "every day"), *screen time* (measured continuously, as the weekly average of the overall amount of time spent watching TV, DVD, video or playing computer games), *physical activity* in the past week (measured continuously, assessing the number of days with at least 60 min of physical activity).

### Family-related variables

Family-related variables included *parental communication* ("good with both parents", "good with mother", "good with father", "difficult with both") and *Family support*. *Family support* was calculated as the average score of four items, measured on a 7-point Likert scale from 1 = "strongly disagree" to 7 = "strongly agree": "My family tries to help", "I get emotional help from my family", "I can talk about problems with my family", "My family helps me with my decisions". The *family support* scale showed good internal consistency (α = 0.89). Thus, higher scores indicated higher support from family.

### School-related variables

School-related variables included *School pressure, Peer support*, *Student support*, and *Teacher support*. *School pressure* was assessed using a single item ("I am stressed by school") on a 4-point Likert scale ranging from 1 = "not at all" to 4 = "a lot". *Peer support* was calculated as the average score of four items, measured on a 7-point Likert scale from 1 = "strongly disagree" to 7 = "strongly agree": "My friends really try to help me", "I can really count on my friends", "I can share my joys and sorrows with my friends", "I can talk about my problems with my friends". The *peer support* scale showed excellent internal consistency (α = 0.90). Thus, higher scores indicated higher support from peers. *Student support* was calculated as the average score of three items, measured on a 5-point Likert scale from 1 = "strongly agree" to 5 = "strongly disagree": "The students in my class enjoy being together", "Most of the students in my class are kind and helpful", "Other students accept me as I am". The *student support* scale showed acceptable internal consistency (α = 0.73). Thus, higher scores indicated a lack of support from students. Finally, *teacher support* was calculated as the average score of three items, measured on a 5-point Likert scale from 1 = "strongly agree" to 5 = "strongly disagree": "I feel that my teachers accept me as I am", "I feel that my teachers care about me as a person", "I feel I can really trust my teachers". The *teacher support* scale showed acceptable internal consistency (α = 0.76). Thus, higher scores indicated a lack of support from teachers.

The same scoring for *family support*, *peer support*, *student support*, and *teacher support*, was applied in other HBSC studies [28, 35].

### Statistical analyses

Statistical analyses were conducted using Jamovi version 2.3.13. General descriptive statistics were computed to describe the sociodemographic characteristics of the participants. Chi-square tests were used to compare non-continuous variables. To explore the determinants of sleep difficulties, we initially tested multilevel logistic models to account for the nesting structure of the data (i.e., classes nested in schools). However, after fitting a null model predicting the odd of sleep difficulties with random-intercept only, we found negligible ICC clustering for classes and schools, suggesting no significant within classes and schools variability for sleep difficulties (ICC < 0.01) [36, 37]. Thus, we used hierarchical logistic regression to calculate the odds ratios (OR) and corresponding 95% confidence intervals (CI) for sleep difficulties. We tested three models: the first model included only control variables and individual-related variables, the second model also included family-related variables, and in the third model, we added the school-related variables. Finally, chi-square tests were calculated to compare the models.

## Results

Table 1 shows the distribution of sleep difficulties among our sample. Overall, 28.3% of adolescents reported having daily sleep difficulties or more than once a week. A significant difference was observed by gender, indicating a higher proportion of sleep difficulties among females (*χ*^*2*^ (1) = 32.66, p < .001).

**Table 1:** Distribution of sleep difficulties among adolescents, overall and according to selected characteristics

	N	(%) No sleep difficulties	(%) Sleep difficulties
Total	3397	71.7	28.3
*Sex:*			
Male	1663	37.3	11.6
Female	1734	34.4	16.6
*Age category:*			
11 years old	1087	23.8	8.2
13 years old	1173	24.6	9.9
15 years old	1137	23.3	10.2
*Family structure*:			
mother and father	2671	58.7	22.2
only mother	399	8.2	3.9
only father	65	1.4	0.3
blended family	133	2.7	1.3
other	41	0.8	0.4
*Siblings*:			
only child	541	12.7	3.9
one sibling	1862	40.7	16.7
at least two siblings	845	18.6	7.4
*Socio-economic status*:			
low	745	15.6	6.9
medium	1638	35.7	13.7
high	931	20.6	7.5

Table 2 shows the ORs and 95% CIs for adolescents' sleep difficulties when considering control and individual-related variables (Model 1). Data showed that females (OR = 1.71; 95% CI: 1.44–2.04) and adolescents with one sibling (OR = 1.37; 95% CI: 1.08–1.75) were more likely than males to have sleep difficulties. Also, increasing alcohol use (OR = 1.16; 95% CI: 1.04–1.29), smoking (OR = 1.12; 95% CI: 1.05–1.21), screen time (OR = 1.06; 95% CI: 1.03–1.09), were associated with an increase likelihood of sleep difficulties. Physical activity was not associated with having or not having sleep problems. The hierarchical logistic regression for Model 1 (Akaike information criterion; AIC = 3392.08) was statistically significant, *χ*^*2*^ (16) = 150.26, *p* =  < 0.001. The model explained 7.0% (Nagelkerke R squared; R^2^_N_) of the variance in sleep difficulties.

**Table 2:** Odds ratios (OR) and corresponding 95% confidence intervals (CI) for sleep difficulties including individual-related variables

**Model 1 predictors**	**Odds ratio**	**Lower CI**	**Upper CI**
*Sex* (male^a^):			
female	1.71***	1.44	2.04
*Age category* (11 years old^a^):			
13 years old	1.05	0.85	1.30
15 years old	0.96	0.76	1.20
*Family structure* (mother and father^1^):			
only mother	1.19	0.92	1.53
only father	0.66	0.31	1.40
blended family	1.14	0.75	1.72
other	1.14	0.54	2.40
*Siblings* (only child^a^):			
one sibling	1.37**	1.08	1.75
at least two siblings	1.31	0.99	1.71
*Socio-economic status* (low^a^):			
medium	0.89	0.72	1.09
high	0.90	0.71	1.14
*Self-perceived ill health*	1.49***	1.3	1.72
*Alcohol use*	1.16**	1.04	1.29
*Smoking*	1.12**	1.05	1.21
*Screen time*	1.06***	1.03	1.09
*Physical activity*	1.00	0.96	1.05

*p < 0.05; **p < 0.01; ***p < 0.001

^a^Reference category

Table 3 shows the ORs and 95% CIs for adolescents' sleep difficulties when adding family-related variables (Model 2). Data showed that Model 1 significant variables were still meaningful other that alcohol use which was not associated with sleep problems. Moreover, adolescents with good communication only with one parent (mothers: OR = 1.30; 95% CI: 1.05–1.62; fathers: OR = 1.71; 95% CI: 1.20–2.43), and difficulties in communication with both parents (OR = 1.75; 95% CI: 1.37–2.25), were more likely to show sleep problems than adolescents reporting overall good parental communication. Also, increasing parental support was associated with a decrease in the odds of sleep problems occurring (OR = 0.82; 95% CI: 0.77–0.78). The hierarchical logistic regression for Model 2 (AIC = 3302.32) was statistically significant, *χ*^*2*^ (20) = 248.02, *p* =  < 0.001. The model explained 12.0% (R^2^_N_) of the variance in sleep difficulties.

**Table 3:** Odds ratios (OR) and corresponding 95% confidence intervals (CI) for sleep difficulties including individual-related, and family-related variables

**Model 2 predictors**	**Odds ratio**	**Lower CI**	**Upper CI**
*Sex* (male^a^):			
female	1.65***	1.37	1.97
*Age category* (11 years old^a^):			
13 years old	0.98	0.79	1.21
15 years old	0.85	0.67	1.08
*Family structure* (mother and father^a^):			
only mother	1.05	0.81	1.36
only father	0.51	0.23	1.11
blended family	1.00	0.65	1.52
other	0.90	0.42	1.92
*Siblings* (only child^a^):			
one sibling	1.34*	1.05	1.71
at least two siblings	1.21	0.91	1.59
*Socio-economic status* (low^a^):			
medium	0.90	0.72	1.11
high	0.93	0.73	1.19
*Self-perceived health*	1.32***	1.14	1.52
*Alcohol use*	1.11	1.00	1.24
*Smoking*	1.12**	1.04	1.20
*Screen time*	1.05***	1.02	1.08
*Physical activity*	1.00	0.96	1.05
*Parental communication* (Good with both parents^a^)			
Good with mother	1.30*	1.05	1.62
Good with father	1.71**	1.20	2.43
Difficult with both	1.75***	1.37	2.25
*Parental support*	0.82***	0.77	0.88

*p < 0.05; **p < 0.01; ***p < 0.001

^a^Reference category

Table 4 shows the ORs and 95% CIs for adolescents' sleep difficulties when adding school-related variables (Model 3). In this model, being in the 15 years old group was associated with a lower probability of sleep problems compared to the 13 years old group (OR = 0.73; 95% CI: 0.57–0.94). Again, Model 2 variables remained significant other than reported good communication only with the mother. Moreover, both increases in school pressure (OR = 1.40; 95% CI: 1.26–1.56) and lack of student support (OR = 1.25; 95% CI: 1.10–1.42) were associated with a higher likelihood of sleep problems, while peer support and teacher support were not. The hierarchical logistic regression for Model 3 (AIC = 3249.10) was statistically significant, *χ*^*2*^ (24) = 309.25, *p* =  < 0.001. The model explained 14.0% (R^2^_N_) of the variance in sleep difficulties.

**Table 4:** Odds ratios (OR) and corresponding 95% confidence intervals (CI) for sleep difficulties including individual-related, family-related, and school-related variables

**Model 3 predictors**	**Odds ratio**	**Lower CI**	**Upper CI**
*Sex* (male^a^):			
female	1.50***	1.25	1.82
*Age category* (11 years old^a^):			
13 years old	0.92	0.74	1.15
15 years old	0.73*	0.57	0.94
*Family structure* (mother and father^a^):			
only mother	1.06	0.81	1.39
only father	0.51	0.23	1.12
blended family	1.05	0.68	1.60
other	1.03	0.48	2.22
*Siblings* (only child^a^):			
one sibling	1.41**	1.10	1.81
at least two siblings	1.28	0.96	1.69
*Socio-economic status* (low^a^):			
medium	0.87	0.70	1.09
high	0.91	0.71	1.16
*Self-perceived health*	1.24**	1.06	1.43
*Alcohol use*	1.10	0.99	1.23
*Smoking*	1.11**	1.03	1.20
*Screen time*	1.05**	1.02	1.08
*Physical activity*	1.00	0.96	1.05
*Parental communication* (Good with both parents^a^)			
Good with mother	1.20	0.96	1.49
Good with father	1.64**	1.15	2.33
Difficult with both	1.63***	1.27	2.10
*Parental support*	0.84***	0.78	0.90
*School pressure*	1.40***	1.26	1.56
*Peer support*	1.05	0.98	1.13
*Student support*	1.25***	1.10	1.42
*Teacher support*	1.04	0.92	1.17

*p < 0.05; **p < 0.01; ***p < 0.001

^a^Reference category

Overall, Model 2 significantly improved over Model 1, *χ*^*2*^ (4) = 97.76, *p* =  < 0.001, and Model 3 significantly improved over Model 2, *χ*^*2*^ (4) = 61.23, *p* =  < 0.001.

## Discussion

This study aimed to provide further evidence on the concurrent contribution of individual and psychosocial determinants of sleep difficulties in adolescence. In line with recent integrated approaches, it explored the simultaneous impact on sleep difficulties of individual (un)healthy behaviors (i.e., alcohol use, smoking, screen time, physical activity), family-related factors (i.e., quality of parental communication, perceived parental support), and school-related factors (i.e., school pressure, peer and student support, and teacher support).

The first objective of our study was to explore the individual-related factors that contribute to sleep difficulties. Our first hypothesis was partially met: indeed, increasing behaviors such as smoking, and screen time enhance the possibility of reporting sleep difficulties, but not alcohol use. Indeed, when considering all psychosocial determinants, alcohol use did not play a significant role in the odds of sleep problems occurring. For, example, in a similar HBSC study conducted in Flanders, alcohol use contributed to sleep difficulties over and beyond psychosocial variables [28]. The result in our sample might be explained by the contribution of other protective factors such as parental support [30, 38]: indeed, based on our findings, a potential cultural aspect within our sample population might arise. Specifically, in Italy, adolescents who perceive their family as reliable sources of support and understanding may be shielded from the negative consequences of maladaptive behaviours, such as excessive alcohol consumption. On the other hand, the absence of perceived parental support may exacerbate issues related to sleep quality, even beyond the effects of alcohol use or misuse. This finding is in line with a review of the literature on the sleeping habits of Italian children and adolescents, which highlighted that more parental control in Italian adolescents was associated with better sleeping habits [39].

Overall, our findings suggest a directly proportional relationship between maladaptive behaviors and the likelihood of sleep problems in adolescence. The available literature emphasizes the impact of "extreme" versions of each behavior (i.e., internet addiction) [8, 10]. However, it seems crucial to consider these phenomena along a dimensional continuum, where even the gradual increase of a specific behavior, not necessarily its pathological version, can significantly impact adolescents' quality of life.

Moreover, our findings showed that increasing physical activity was not associated with sleep difficulties. In line with the contribution of Ghekiere and colleagues (2019), we can hypothesize that, contrary to other studies, this finding could be related to the way the HBSC protocol assesses physical activity [12, 13]. Indeed, by relying only on a self-report estimate of how many days per week adolescents enroll in at least 60 min of physical activity (according to the WHO suggestions for children and adolescents aged 5–17 years), HBSC data might not consider more detailed aspects of physical activity such as intensity, actual duration, and the time of day when the activity is performed. Similar results were found in other HBSC studies [12, 14, 28].

The second objective of our contribution was to consider the impact of family-related variables, meaning the quality of adolescents' communication with their families and their perceived parental support. In line with our second hypothesis, findings suggest that the quality of communication with parents matters. Indeed, the study findings emphasize the importance of good communication with both parents for adolescents. This data aligns with the relevant literature and adds that good communication should occur with both parents, not just one of them [40, 41]. In addition, perceived parental support emerges as another significant factor that suggests the importance for adolescents to feel emotionally understood and to perceive the possibility of opening up and talking about their problems with their parents. The family context is often linked to the physical context of adolescents' sleep: the study findings support the assumption that a psychologically stable and available context is also associated with the possibility of fewer sleep problems [30]. Previous Italian HBSC findings highlighted the importance of parental support as a protective factor against health complaints [42]. Moreover, these results are in line with other HBSC studies conducted in Greece and in Sweden that highlighted the inverse association between parental support and health complaints [43, 44].

The third objective of our contribution was to consider the impact on sleep of school-related variables, meaning adolescents’ reported school pressure and perceived support from their peers, schoolmates/students, and teachers. Interestingly, literature usually does not differentiate between peers (i.e., friends outside the school context) and students/schoolmates (i.e., peers inside the school context): however, findings showed that, contrary to available evidence, an increase in perceived support from peers is not associated with a lower chance of sleep problems. In contrast, perceived support from schoolmates has a significant impact. Again, these findings can be explained by the specific HBSC items measuring student support that seem to focus on the dimension of acceptance (i.e., "Other students accept me as I am") and the items measuring peer support, that seems to focus on the dimension of availability and help (i.e., "My friends really try to help me"). Data, therefore, suggest that the relationship with peers has an impact on sleep quality when the quality of relationship expresses the adolescents’ feeling of inclusion and acceptance among their peers (student support) and not their availability to ask for help (peer support), which is something adolescents’ might expect from adult figures instead (parental support) [38]. Notably, another HBSC research on a Scottish sample highlighted the centrality of the school context in adolescence exploring the role of school connectedness (i.e., how much students feel connection and attachment to their classmates and teachers) as a determinant of adolescents’ well-being [45]. Overall, numerous evidence underline that feeling accepted by peers in adolescence is a significant predictor of well-being [46] and is also consistent with the increasing sensitivity to social judgment to which adolescents' social brains are subjected [47]. Again, this finding is also in line with a similar study conducted on another HBSC sample from Flanders [28].

In addition, findings showed no association between perceived teacher support and sleep quality. Differently, other HBSC studies highlighted that perceived teacher support was associated with lower odds of health complaints [43, 48]: thus, our finding might highlight a specificity of the Lombardy sample that may be explained by the greater weight of the quality of their relationship with their schoolmates (i.e., acceptance) and a different role attributed to teachers (i.e., motivation, engagement) [21]. Also, in line with other contributions, school pressure's impact is a factor promoting sleep problems [49]. In this scenario, it seems that within the school context, each actor exerts a different role: on the one hand, schoolmates contribute to the sense of acceptance that is crucial at this stage of development; on the other hand, the school environment may foster a sense of demand that, if perceived as excessive, can become problematic for adolescents.

This study's results should be understood in the context of the study’s limitations. First, the study design is cross-sectional, thus, no causal inference can be made between the variables considered. Second, all variables considered in the research were collected via self-report questionnaires: despite the evident practicality in extensive data collections and school environments, future studies should include different methodologies for a more fine-grained evaluation of sleep difficulties (i.e., actigraphy, daily diaries). Third, the current findings must be replicated in culturally diverse populations (i.e., HBSC data collections in other countries).

In conclusion, our contribution underlines the importance of studying concurrent individual, familial and school-related determinants of sleep difficulties in adolescence, a phenomenon that, in our sample, affects more than one adolescent out of four. Indeed, it adds evidence to the importance of an integrated approach to the study of sleep difficulties in adolescence, including psychosocial variables. In this scenario, it is critical to account for family-related aspects, such as the quality of parental communication and perceived parental support, as expressions of adult figures' emotional and behavioral availability in the adolescent’s life. Also, findings highlight the importance of considering the quality of the day-to-day relationship with schoolmates and the school level of demands.

All in all, these findings, highlighting specific psychosocial contributors to sleep difficulties, support further reflections to benefit both crucial stakeholders (i.e., family, school) in adolescents’ lives as well as clinical practice and intervention. More, the context of these findings is essential for interpreting the study results. For example, understanding the cultural norms and values influencing health behaviours in Italy can help researchers and policymakers develop effective strategies for promoting healthy lifestyles among young people. Thus, acknowledging these cultural factors (i.e., the role of parental and schoolmates' support) will be essential when designing interventions to address health issues among school-aged children in Italy.

## Data Availability

The datasets generated during and/or analysed during the current study are available from the corresponding author upon reasonable request.
